# Molecular Analysis of the Contribution of Alkaline Protease A and Elastase B to the Virulence of *Pseudomonas aeruginosa* Bloodstream Infections

**DOI:** 10.3389/fcimb.2021.816356

**Published:** 2022-01-12

**Authors:** Margalida Mateu-Borrás, Laura Zamorano, Alex González-Alsina, Irina Sánchez-Diener, Antonio Doménech-Sánchez, Antonio Oliver, Sebastián Albertí

**Affiliations:** ^1^ Instituto Universitario de Investigación en Ciencias de la Salud (IUNICS), Universidad de las Islas Baleares, Palma de Mallorca, Spain; ^2^ Instituto de Investigación Sanitaria Illes Balears (IdISBa), Palma de Mallorca, Spain; ^3^ Unidad de Investigación, Hospital Son Espases, Palma de Mallorca, Spain; ^4^ Servicio de Microbiología, Hospital Son Espases, Palma de Mallorca, Spain

**Keywords:** alkaline protease, elastase B, complement component C3, bloodstream infection, *Pseudomonas aeruginosa*

## Abstract

*Pseudomonas aeruginosa* is a major cause of nosocomial bloodstream infections. This microorganism secretes two major proteases, alkaline protease A (AprA) and elastase B (LasB). Despite several *in vitro* studies having demonstrated that both purified proteases cleave a number of components of the immune system, their contribution to *P. aeruginosa* bloodstream infections *in vivo* remains poorly investigated. In this study, we used a set of isogenic mutants deficient in AprA, LasB or both to demonstrate that these exoproteases are sufficient to cleave the complement component C3, either soluble or deposited on the bacteria. Nonetheless, exoprotease-deficient mutants were as virulent as the wild-type strain in a murine model of systemic infection, in *Caenorhabditis elegans* and in *Galleria mellonella*. Consistently, the effect of the exoproteases on the opsonization of *P. aeruginosa* by C3 became evident four hours after the initial interaction of the complement with the microorganism and was not crucial to survival in blood. These results indicate that exoproteases AprA and LasB, although conferring the capacity to cleave C3, are not essential for the virulence of *P. aeruginosa* bloodstream infections.

## Introduction

*Pseudomonas aeruginosa* is one of the most common Gram-negative organisms causing nosocomial bacteremia. Despite improvements in antimicrobial therapy and hospital care, *P. aeruginosa* bacteremia remains fatal in about 30% of cases (Peña et al., 2012; Peña et al., 2013). The reasons for the mortality of *P. aeruginosa* bacteremia are multifactorial and include the intrinsic virulence of the microorganism and some underlying host conditions (Chamot et al., 2003; Kang et al., 2005; Peña et al., 2015). It is remarkable that a large number of deaths occur within the first 24–72 hours after *P. aeruginosa* infection (Hattemer et al., 2013; Peña et al., 2013), suggesting an inefficient function of the early innate immune mechanisms of the host.

Complement is the main early innate immune effector in the blood. Clinical and experimental evidence demonstrates that this system is critical in host defense against *P. aeruginosa* (Mueller-Ortiz et al., 2004; Dahlke et al., 2011; Khan et al., 2018). C3 is the most abundant complement protein within serum. It is a 190 kDa protein, composed of two glycoprotein chains (α and β) associated through disulphide bonds. It is required for each of the three complement-activating pathways: classical, lectin, and alternative. It has an essential role in the clearance of the microorganism because it is the source of the phagocyte chemoattractant factor C3a and of fragments such C3b, that facilitates bacterial recognition by phagocytes. In addition, C3b is also involved in the formation of C5 convertases that cleave C5 and trigger the subsequent deposition of the lytic pathway complement components to form the membrane attack complex, which may cause the killing of this microorganism.

*P. aeruginosa* secretes two major proteases, the 33-kDa elastase LasB and the 50-kDa alkaline protease AprA. *In vitro* experiments using purified protease have shown that LasB degrades host immune components including surfactant proteins (Alcorn and Wright, 2004) and complement component C3 (Schultz and Miller, 1974; Hong and Ghebrehiwet, 1992), which collectively function to promote bacterial clearance. Furthermore, *in vivo* experiments have demonstrated that LasB is important for the establishment of *P. aeruginosa* respiratory infection (Kuang et al., 2011; Bastaert et al., 2018). Surprisingly, little is known about the role of LasB in *P. aeruginosa* bloodstream infections.

On the other hand, *in vitro* studies using purified AprA have shown that this protease impedes bacterial clearance by degrading C3 (Hong and Ghebrehiwet, 1992), C1s and C2 (Laarman et al., 2012), thereby preventing complement-mediated phagocytosis. However, the impact of AprA deficiency on *P. aeruginosa* virulence remains poorly investigated.

In the present study, we used a set of isogenic mutants deficient in AprA, LasB or both to analyze the contribution of both proteases to the virulence of *P. aeruginosa* in a murine model of systemic infection.

## Materials and Methods

### Bacterial Strains

*P. aeruginosa* reference strain PA14 and its isogenic *aprA-*deficient mutant (PA14Δ*aprA*), *lasB-*deficient mutant (PA14Δ*lasB*) and *aprA* and *lasB-*deficient double mutant (PA14Δ*aprA*Δ*lasB)* were previously described (Casilag et al., 2015). RT-PCR analysis of the specific *aprA* and *lasB* transcripts and mass spectrometry analysis of the proteins present in the cell-free supernatant of the bacterial cultures confirmed the phenotype of each strain.

Bacterial cells were grown in LB broth or in human serum at 37°C with shaking or in LB solidified with 1.5% agar.

### Human Serum

Blood samples were collected at Hospital Son Espases (Palma de Mallorca, Spain) from 6 healthy individuals. Blood was centrifuged at 1500 ×g for 20 min. to separate serum. Equal volumes of serum from each individual was mixed to get a pool that was aliquoted and stored at −80°C until its use.

### C3 Cleavage Assays

To study the cleavage of C3 by *P. aeruginosa* we used the cell-free supernatant of bacterial cultures grown overnight at 37°C in LB broth. The culture supernatant and bacterial cells were separated by centrifugation and then *via* filtration through a 0.22-μm-pore-size membrane (Millipore).

Purified human C3 (300 ng) (Sigma-Aldrich) or human serum diluted in phosphate buffered saline (PBS) (10% final concentration), was incubated with the cell-free supernatant of bacterial cultures or LB as control for 3 h at 37°C. Samples were boiled in loading buffer and resolved by Sodium-Dodecyl Sulfate-PolyAcrylamide Gel Electrophoresis (SDS-PAGE). Separated proteins were transferred onto Immobilon-P membranes which were blocked for 1 h at room temperature with 1% bovine serum albumin (BSA) in PBS and incubated overnight at 4°C with rabbit polyclonal anti-human C3 (Abcam). The membranes were washed with PBS and incubated 1 h at room temperature with alkaline phosphatase-conjugated secondary antibody (Sigma-Aldrich). Finally, membranes were developed with the Fast 5-bromo-4-chloro-3-indolyl phosphate–nitroblue tetrazolium kit (Sigma-Aldrich).

For the N-terminal sequencing, C3 (5 μg) was incubated with the cell-free supernatant of an overnight PA14 culture. Cleavage products were resolved by SDS-PAGE, excised and analyzed by N-terminal sequencing, as previously described (Lynskey et al., 2017).

### C3 Deposition Assays

Approximately 1x10^9^ CFU were washed with PBS and opsonized 1 h at 37°C with human serum, human serum pre-treated 1h at 37°C with cell-free supernatants from bacterial cultures or heat inactivated serum (30 min at 56°C), as control. All sera were diluted in PBS (25% final concentration). Next, bacterial cells were washed with PBS and incubated 2 h at 37°C in 50 mM carbonate-bicarbonate buffer (pH 9.0) containing 1 M NH_4_OH to release C3 fragments bound to the bacterial surface. Finally, cell-bound C3 was quantified by ELISA, as previously described (Qadi et al., 2017).

### Murine Model of Systemic Infection

Mouse lethality studies were performed with female CD1 mice (16 -20 g of weight) (Harlan Ibérica, S.L.). Mice (n = 7 per group) were challenged *via* intraperitoneal injection with 5 x 10^5^ CFU of *P. aeruginosa* and monitored every 12 h during 72 h. Bacteremia was evaluated every 12 h by plating 10-30 μl of tail vein blood on LB agar plates. Lethality studies were also performed in mice with reduced number of neutrophils in blood (neutropenic). Neutropenia was induced by intraperitoneal injection of cyclophosphamide (Sigma) 150, 100, 100 mg/kg of body weight on day 1, 3, and 4, respectively. Cell counting confirmed that cyclophosphamide reduced by 90% the number of polymorphonuclear cells in blood. One day after the last injection, mice (n = 10 per group) were infected with *P. aeruginosa* (2 x 10^2^ CFU) and monitored daily during 4 days.

### *Caenorhabditis elegans* Virulence Assays

The virulence assays in the *C. elegans* model were conducted using the protocol described by Sánchez-Diener et al. (Sánchez-Diener et al., 2017). Briefly, fresh bacterial cultures were spread onto potato dextrose agar plates and incubated at 37°C for 24 h to form bacterial lawns. Five worms per plate were poured on top of the bacterial lawns. The plates were incubated at 24°C and observed at 20 and 40 magnifications to detect the presence of living worms at 0 h, 24 h, 72 h, and 168 h. A worm was considered dead when it no longer responded to touch.

A worm was considered dead if it did not move spontaneously. At least three independent experiments were performed for each strain. Thus, a total number of 15 worms per strain were used.

### *Galleria mellonella* Virulence Assays

The virulence assays in the *G. mellonella* model were performed as previously described (Pérez-Gallego et al., 2016). *G. mellonella* were purchase from a local supplier. Briefly, 10 microliters containing 10, 5 or 2 CFU of bacterial exponential cultures, were injected into individual fifth-instar *G. mellonella* larvae (n = 10 per dose and strain) *via* the hindmost left proleg. Survival rate was assessed after 24 h at 37°C. A lethal dose (LD_50_) was obtained from three independent experiments including a control of 10 larvae challenged with 10 microliters of PBS in each experiment. LD50 was calculated using the Probit´log(dose) regression model, a type of regression used to analyze binary outcome variables. (Finney, 1967).

### RNA Extraction and Real-Time PCR

Total cellular RNA was isolated from *P. aeruginosa* using the Qiagen Rneasy Mini Kit (Qiagen) according to the manufacturer’s instructions. Contaminating DNA was removed using Turbo DNA-*free* kit (Thermo Fisher). After DNase treatment, one step reverse transcription and real-time PCR amplification was performed on 50 ng of purified RNA by using the QuantiTect SYBR green RT-PCR kit (Qiagen) with an Eco Real-Time PCR System. Negative controls consisting of RNase-free water were added to detect possible DNA contamination. Data were analyzed with the EcoStudy Software V.5.0. The mRNA of *aprA* and *lasB* genes was normalized using the housekeeping gene *rpsl* as a reference. Primers used for the amplification of *aprA, lasB* and *rpsl* are described in **Supplementary Table S1**. The level of gene expression was calculated using comparative 2^-Ct method (Livak and Schmittgen, 2001).

### Serum and Whole Blood Resistance Assays

For serum resistance assays, approximately 1x10^9^ CFU were incubated at 37°C in a pool of human sera diluted in PBS (25% final concentration). At different time points, survival was determined by counting colonies on LB agar plates.

For whole blood resistance assays, approximately 2.5x10^7^ CFU resuspended in RPMI-1640 were incubated at 37°C in 1 ml of heparinized blood (90% final concentration) from several healthy donors. The multiplicity of infection was 5 bacteria per phagocyte. Bacterial quantitative cultures were performed at different times by plating on LB agar plates.

### Statistical Analysis

The statistical significance of the data obtained in at least three independent experiments was determined by ANOVA with *post hoc* Tukey. In all cases, a p value of <0.05 was considered statistically significant. The log rank test was used for survival analysis. LD50s were determined using a Probit model. ANCOVA analysis was used in **Figure 3B**. Statistical analyses were performed using IBM SPSS Statistics v22 software.

### Ethics Statements

All animal experiments were approved by the Ethics Committee of Animal Research of Islas Baleares [Comité Ético de Experimentación Animal UIB (CEEA-UIB)] and conducted in accordance with the institutional and national guidelines and regulations.

All human samples were taken after obtaining the “Informed consent” of the participants. They had been informed of the purposes of the study. The study was reviewed and approved by the Ethics Review Committee of Human Experimentation of Islas Baleares [Comité de ética de la Investigación de las Islas Baleares (CEI-IB)].

All study procedures were performed in accordance with relevant standard international guidelines/regulations.

## Results

### Exoproteases AprA and LasB Are Sufficient to Cleave C3

Previous *in vitro* studies have shown that purified alkaline protease AprA and LasB elastase degrade C3 (Hong and Ghebrehiwet, 1992; Laarman et al., 2012). In order to determine whether these are the unique*P. aeruginosa* exoproteases involved in the cleavage of C3, the cell-free supernatant of bacterial growth cultures from the wild-type strain PA14 and the isogenic strains that differed only in the production of AprA (PA14Δ*aprA*), LasB (PA14Δ*lasB*) or both proteases (PA14Δ*aprA*Δ*lasB*) were incubated for 3 h with human purified C3.

**Figure 1:**
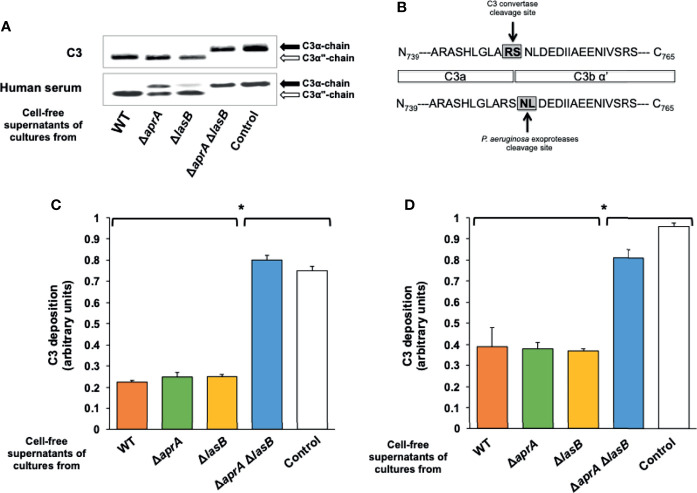
P*. aeruginosa* exoproteases AprA and LasB cleave C3. **(A)** Exoproteases AprA and LasB cleave human C3. Representative Western blot analysis of C3 cleavage by *P. aeruginosa*. Purified human C3 (300 ng) or human serum (10%) were incubated for 3 h at 37°C with LB (control) or the cell-free supernatants obtained from cultures of the wild-type (WT) *P. aeruginosa* PA14 and the isogenic deficient mutants in AprA (Δ*aprA)*, LasB (Δ*lasB)* or both (Δ*aprA* Δ*lasB*). Proteins were separated by SDS-PAGE and subjected to a Western blot with a rabbit polyclonal antibody anti-C3. Specific cleavage of the C3α chain (black arrow) resulted in release of a ≈100 kDa product, C3α” (white arrow). Full-length blots are presented in **Supplementary Figure S1**. **(B)** C3 cleaving site of exoproteases is different to the C3 convertase cleavage site. C3α” was subjected to N terminal sequencing. Exoproteases was demonstrated to cleave C3 between Asp^751^ and Leu^752^, 2 amino acids downstream to the physiological C3 convertase cleavage site. **(C, D)** Exoproteases AprA and LasB cleave C3 either soluble or deposited on the bacteria. Analysis of complement component C3 deposition on *P. aeruginosa*. In C, cells of the wild-type strain PA14 were incubated 1h in human serum pre-treated (1h at 37°C) with the cell-free supernatants of cultures from the wild-type *P. aeruginosa* PA14 (WT) or from the isogenic deficient mutants in AprA (Δ*aprA)*, LasB (Δ*lasB)* or both (Δ*aprA* Δ*lasB*), while in D, cells of the wild-type strain were incubated 1h in human serum, washed and then treated with the cell-free supernatants (2h at 37°C). C3 deposited on the bacterial surface was determined by ELISA. Control values in heat inactivated serum were always < 0.1 arbitrary units and were subtracted from the values obtained with human serum. Data represents three experiments done in duplicate. Errors bars represent SEMs. Statistical analyses were performed using ANOVA with *post hoc* Tukey; *P < 0.05.

Under reducing conditions, human C3 migrates as two separate bands of 111-kDa (α chain) and 75-kDa (β chain). Incubation of the cell-free supernatants of cultures from PA14 or the mutants PA14Δ*aprA* and PA14Δ*lasB* with C3 generated a ~100-kDa α-chain (α”-chain) resulting from cleavage of the 111-kDa α chain (**Figure 1A**), while the β chain remained intact (**Supplementary Figure S1**). This α”-chain was absent from the samples incubated with the cell-free supernatant of the culture from the double mutant or the control incubated with LB alone, showing that the presence of this chain was due to the *P. aeruginosa-*mediated cleavage of C3 rather than to the physiological activation of C3, which also would result in the formation of a smaller α-chain (α’). Furthermore, N-terminal sequencing of the α”-chain demonstrated that *P. aeruginosa* exoproteases cleave C3 between Asp^751^ and Leu^752^, 2 amino acids upstream of the physiological C3 convertase cleavage site (**Figure 1B**).

We conducted a similar experiment but using human serum as source of C3 instead of purified C3 (**Figure 1A**). The cell-free supernatant of the culture from the double mutant was unable to cleave C3, suggesting that AprA and LasB are the main exoproteases of *P. aeruginosa* with the capacity to cleave C3. The cell-free supernatant of the culture from the AprA-deficient mutant was impaired in its ability to cleave C3 present in the human serum, while that of the LasB-deficient mutant (PA14Δ*lasB*) was able to cleave C3 almost as efficiently as the cell-free supernatant from the wild-type strain. These observations indicate that both proteases are sufficient to cleave C3. However, AprA apparently cleaves C3 from serum more efficiently than LasB.

We determined the opsonization of *P. aeruginosa* by human serum pre-treated 1h at 37°C with the cell-free supernatants of the overnight cultures from PA14 and the isogenic mutants (**Figure 1C**). Consistent with the results described above, the pre-treatment of human serum with the cell-free supernatants of cultures from PA14, PA14Δ*aprA* or PA14Δ*lasB* similarly reduced C3 deposition on *P. aeruginosa*. Conversely, the serum pre-treated with the cell-free supernatant of the culture from the double mutant opsonized *P. aeruginosa* as efficiently as the untreated serum. We also tested the ability of both proteases to degrade C3 deposited on the bacteria. In this experiment, bacterial cells of the parent strain PA14 were opsonized with human serum, washed and treated for 2 h at 37°C with different cell-free supernatants. The cell-free supernatants of cultures from PA14 and the single mutants PA14Δ*aprA*, PA14Δ*lasB* reduced the amount of opsonizing C3 deposited on *P. aeruginosa* similarly, whereas the cell-free supernatant of the culture from the double mutant PA14Δ*aprA*Δl*asB* had no effect (**Figure 1D**).

Altogether, these results demonstrate that both AprA and LasB must be deleted in order to reduce C3 opsonization and that either protease is sufficient to reduce this activity.

### AprA and LasB Are Not Essential for the Virulence of *P. aeruginosa*

**Figure 2:**
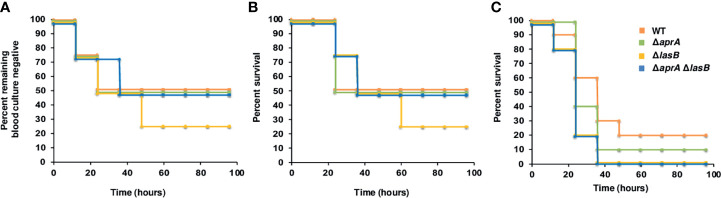
Impact of AprA and LasB on the virulence of *P. aeruginosa* in a murine model of systemic infection. Analysis of time to first positive blood culture in healthy mice **(A)** and survival curves over 4 days of healthy mice (n = 7) **(B)** or neutropenic mice (n = 10) **(C)** infected with 5 x 10^5^ CFU **(B)** or 2 x 10^2^ CFU **(C)** of *P. aeruginosa* WT strain PA14 (orange), or its derived isogenic mutants PA14Δ*aprA* (green), PA14Δ*lasB* (yellow) and PA14Δ*aprA*Δ*lasB* (blue). The time to first positive culture and the difference in survival between the groups were not significantly different by log rank test.

To investigate the impact of AprA, LasB or both in the pathogenesis of *P. aeruginosa* bloodstream infections, we tested the capacity of the PA14Δ*aprA*, PA14Δ*lasB* and PA14Δ*apraA*Δ*lasB* mutants to cause bacteremia and lethal infection in a murine model of systemic infection. All mutants showed *in vitro* growth rates similar to the parent strain. Mice were infected intraperitoneally with 5 x 10^5^ CFU of the parent strain PA14 or the isogenic mutants and monitored for the development of a positive blood culture. In total, 50–25% of the animals developed bacteremia before 20 h, independent of the strain used for the infection (**Figure 2A**). The analysis of survival indicated that bacteremia preceded fatal infection by 12–24 h, and 75–50% of the animals infected with any strain died, with the majority of deaths occurring before 36 h and without significant differences between strains (**Figure 2B**). We did not observe significant differences in the ability to cause lethal infection between strains when we used a higher (10^6^ CFU) or lower infective dose (10^4^ CFU) (data not shown).

Given that neutropenia is a frequent predisposing condition of *P. aeruginosa* bloodstream infections, we also tested the effect of both exoproteases in a neutropenic model of systemic infection. As shown in **Figure 2C**, the mutant strains in this model were as virulent as the parent strain PA14.

Analysis of the organisms isolated from blood or spleen of infected animals confirmed the original mutant phenotype, indicating that all mutants were fully virulent in mice and that virulence was not due to a reversion to the wild-type phenotype.

To further extend these results we explored whether these exoproteases had an impact on virulence of *P. aeruginosa* in two well-established infection models: *C. elegans* (Feinbaum et al., 2012) and *G. mellonella* (Jander et al., 2000). As shown in **Supplementary Figure S2**, the inactivation of *aprA*, *lasB* or both did not reduce the capacity of *P. aeruginosa* strain PA14 to kill *C. elegans* (panel A) or *G. mellonella* (panel B).

**Figure 3:**
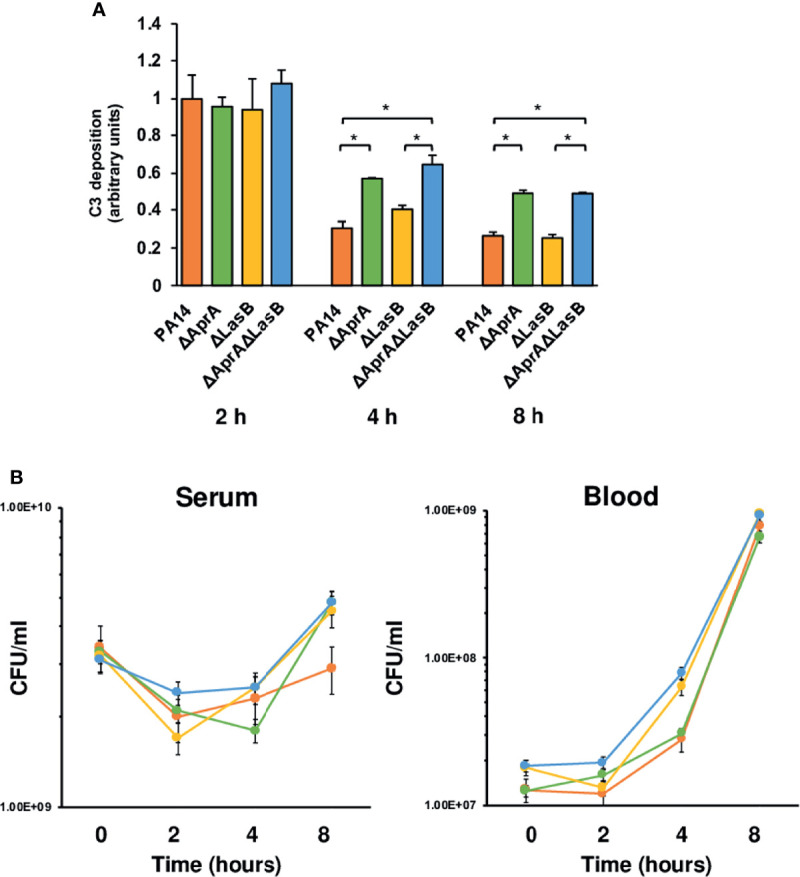
Impact of AprA and LasB on the survival of *P. aeruginosa* in human serum or blood. **(A)** Analysis of complement component C3 deposition on *P. aeruginosa*. Wild-type strain PA14 or the isogenic mutants deficient in AprA, LasB or both were incubated in human serum and C3 deposited on the bacterial surface was determined by ELISA at different times. Control values in heat inactivated serum were always < 0.1 arbitrary units and were subtracted from the values obtained with human serum. Data represents three experiments done in duplicate. Errors bars represent SEMs. Statistical analyses were performed using ANOVA with *post hoc* Tukey; *P < 0.05. **(B)** Survival of *P. aeruginosa* strains in human serum or whole blood. *P. aeruginosa* WT strain PA14 (orange), or its derived isogenic mutants PA14Δ*aprA* (green), PA14Δ*lasB* (yellow) and PA14Δ*aprA*Δ*lasB* (blue) were incubated in human serum or whole blood and quantitative bacterial cultures were performed at different times by plating on LB agar plates. Data represents three experiments done in duplicate. Errors bars represent SEMs. Statistical analyses were performed using ANCOVA.

The results described above present an apparent paradox: AprA and LasB cleaves C3 *in vitro* and reduces *P. aeruginosa* opsonization by C3. However, the results of the murine model of systemic infection suggest that AprA or LasB deficiency does not attenuate the virulence of the microorganism. A reasonable explanation for this paradox is that complement deposition occurs rapidly *in vivo* before *P. aeruginosa* expresses sufficient AprA or LasB to degrade C3. Therefore, the microorganism uses other virulence factors to evade complement-mediated killing before exoproteases are available. To test this hypothesis, we quantified the deposition of human complement component C3 onto PA14 and the isogenic mutants grown in human serum at different times (**Figure 3A**). We observed a decrease of C3 deposited onto the bacteria over the time, regardless of the C3-cleaving phenotype caused by the consumption of the complement system components induced by the bacterial cells (Schiller and Joiner, 1986). After 2 h of incubation, all strains bound similar amounts of C3. The C3-cleaving activity of AprA became evident after 4 h of incubation and was manifested by a significant reduction of the amount of C3 deposited onto the parental strain PA14 and the LasB deficient mutant that expresses AprA. Therefore, only after more than 2 h does *P. aeruginosa* produce sufficient AprA to mitigate the deposition of C3. This finding correlated well with the course of *aprA* expression in human serum (**Supplementary Figure S3**). The relative quantification of *aprA* and *lasB* transcripts demonstrated that, at each time point, the transcriptional rate of *lasB* was remarkably lower than *aprA*. Maximal transcription of both genes in human serum was observed at 4 h and then decreased and stabilized at 8 h.

To test whether C3 degradation had any influence on the capacity of *P. aeruginosa* to grow in human serum, where the complement system is the main effector, we evaluated the viability of PA14 and the isogenic mutants in an ex vivo serum model of infection. Fresh human serum was inoculated with each strain and the total number of viable bacteria was determined at different times by counting colonies on LB agar plates. Comparison of the quantitative serum culture results for the four bacterial strains reveals that the incapacity to cleave C3 did not impair the ability to survive in serum (**Figure 3B**, left panel). Thus, all strains exhibited similar growth curves independently of their C3 cleaving phenotype.

C3 derived fragments deposited onto the bacteria, such as C3b, facilitate bacterial recognition and killing by phagocytes. To evaluate the impact of the C3-cleaving phenotype on the resistance of *P. aeruginosa* to the complement-mediated phagocytic killing, we used an ex vivo model of infection, but using blood, that contains phagocytic cells, instead of serum. All strains displayed similar growth patterns in human blood, suggesting the cleavage of C3 does not contribute significantly to the capacity of *P. aeuginosa* to grown in blood. In consistence with this observation, the number of bacteria recovered from the blood of mice after challenge with the wild-type strain was similar to that recovered from mice infected with isogenic mutants (**Supplementary Figure S4**).

These results provide strong support to the idea that although the exoproteases AprA and LasB, mainly AprA, reduce the deposition of C3, they are not essential for *P. aeruginosa* to survive in the bloodstream.

## Discussion

Elastase B and alkaline protease A are the main proteases secreted by *P. aeruginosa*. The capacity of both proteases to degrade or inactivate C3, an essential complement component for the host early immune response, has been well documented *in vitro* using purified proteins (Schultz and Miller, 1974; Hong and Ghebrehiwet, 1992).

In the present study, we used a microbiological approach, instead of purified proteins, to complement previous results and provide new relevant information. Thereby, using a set of isogenic mutants deficient in AprA, LasB or both, we demonstrate that these proteases are sufficient to cleave soluble C3 and the opsonic C3 fragments deposited on the bacterial surface. Our findings indicate that both exoproteases are expressed efficiently in human serum, which is consistent with the transcriptome analysis conducted by Turner et al. (Turner et al., 2014) showing that both, *aprA* and *lasB* were upregulated in the host. Furthermore, we demonstrate that the C3-cleaving activity of LasB in serum is almost residual compare to AprA, may be because LasB cleaves preferentially other substrates present in the human serum (Schultz and Miller, 1974).

The proteolytic activity of both AprA and LasB resulted in slight reduction of the apparent molecular weight of the 111-kDa α chain. In contrast, Hong et al. (Hong and Ghebrehiwet, 1992) reported the complete disappearance of the 111-kDa α-chain concomitant with the appearance of two prominent α-chain fragments with apparent molecular weights of approximately 41 and 26 kDa. It is likely that this discrepancy may be attributed to the shorter digestion time used in our study (3h vs 20h). In fact, N-terminal sequencing of the resulting C3α” fragment revealed that the C3 cleaving site of *P. aeruginosa* exoproteases AprA and LasB is identical to that reported for aureolysin from *Staphylococcus aureus* (Laarman et al., 2011) or GelE from *Enterococcus faecalis* (Park et al., 2008). Interestingly, these exoproteases generate a smaller C3b in contrast to the C3-cleaving proteases anchored to the outer surface of the microorganisms, such as ScpA in *Streptococcus pyogenes* (Lynskey et al., 2017) or NalP in *Neisseria meningitidis* (Del Tordello et al., 2014), which generate a longer C3b fragment.

In this manuscript, we have used three different models to test the role of AprA and LasB in the virulence of *P. aeruginosa.* Our results clearly demonstrate that AprA is not essential for the virulence of *P. aeruginosa*. Conversely, at least two previous reports evaluated the contribution of LasB to lung infection by *P. aeruginosa* (Kuang et al., 2011; Bastaert et al., 2018). In contrast with our results, both studies concluded that LasB deficiency attenuated the virulence of *P. aeruginosa* PAO1 during lung infection. Several nonexclusive factors might explain this apparent discrepancy. First, in our study we used the clinical isolate *P. aeruginosa* PA14, which is more virulent than PAO1 (Rahme et al., 1995; Tan et al., 1999; Choi et al., 2002). Furthermore, as the genomic analysis of both strains revealed, the genes involved in the pathogenicity of PAO1 are not necessarily relevant for or predictive of the virulence of PA14 (Lee et al., 2006). Second, in the previous studies, mice were infected with 100-fold (Kuang et al., 2011) and 10,000-fold (Bastaert et al., 2018) more bacteria than in our study, which probably led to the higher production of LasB by the wild-type strain.

Our experiments performed *in vitro* clearly demonstrated that the mutants deficient in AprA, LasB or both were indistinguishable from the wild-type strain in terms of binding of C3 at least in the first 2 h. Similarly, Kuang et al. demonstrated that *P. aeruginosa* PAO1 needed at least 6 hours to produce enough LasB elastase to slightly reduce the amount of intact surfactant protein A (Kuang et al., 2011).

As other virulence factors, the production of AprA and LasB is controlled according to the cell density by signal molecules that are released into the medium and sensed by the quorum sensing system las, which, *via* the transcriptional activator LasR, induce the expression of *aprA* and *lasB.* These signal molecules are released into the medium, where they accumulate as cells grow to high densities and reach a “quorum” (Smith and Iglewski, 2003). Therefore, it is not surprising that it takes a substantial period of time to observe the effects of both exoproteases on C3.

This finding indicates that *P. aeruginosa* expresses other virulence factors to evade the complement attack before the exoproteases are released. Thus, the observation that the ability of the isogenic deficient-exoprotease deficient mutants to survive in blood is similar to the parental strain is a probably a consequence of redundancy of the multiple virulence factors produced by this pathogen and reflects that disease severity is multifactorial and context dependent, a fact that might complicate our efforts to identify the key virulence factors in each case (Granato et al., 2016).

According to our results, it is likely that AprA and LasB contribute to the virulence of *P. aeruginosa* once the infection is already established, but not in the early steps when the innate immune system is faster than the synthesis of both proteases by the microorganism. Thus, both exoproteases might facilitate the dissemination of the microorganism, disrupting the endothelial barrier from the initial focus of the infection (Golovkine et al., 2014), as in pneumonias, or participate in the modulation of the inflammatory response in the chronic respiratory infections (LaFayette et al., 2015; Hennemann et al., 2021).

Our findings and conclusions challenge some established dogmas with regards to the role of the C3-cleaving activity of some proteases in the bacterial virulence. Many pathogens secrete proteases with the capacity to degrade C3 including *S. aureus* (Aureolysin) (Laarman et al., 2011), *E. faecalis* (GelE) (Park et al., 2008) and pathogenic *Leptospira* (Thermolysin metalloproteinase) (Rodrigues et al., 2014), among others. In most of these cases, the cleaving activity has been tested *in vitro* using purified proteases or the supernatants of the bacterial cultures, but their contribution to virulence has not been tested *in vivo*. According to our results, the C3-cleaving activity mediated by those proteases might not be essential for the virulence of those microorganisms. In contrast, the C3-cleaving proteases anchored to the outer surface of the microorganisms, such as ScpA in *S. pyogenes* (Lynskey et al., 2017) or NalP in *N. meningitidis* (Del Tordello et al., 2014) are ready to act immediately and play a key role in the virulence of these microorganisms. In fact, both, the ScpA or the NalP deficient mutants were attenuated *in vivo* or in human serum, respectively.

In summary, our results indicate that AprA and LasB confer the capacity to degrade C3 to *P. aeruginosa*, but this cleaving activity is not essential for the virulence of the microorganism.

## Data Availability Statement

The original contributions presented in the study are included in the article/**Supplementary Material**. Further inquiries can be directed to the corresponding author.

## Ethics Statement

The animal study was reviewed and approved by Comité Ético de Experimentación Animal UIB (CEEA-UIB).

## Author Contributions

SA conceived the study. MM-B, LZ, IS-D, and AG-A carried out the experiments and analyzed data. AD-S, AO, and SA contributed to experimental design and the interpretation of results. SA and AO supervised the project. SA wrote the manuscript with support from MM-B. All authors contributed to the article and approved the submitted version.

## Funding

This work was supported by grant RTI2018-100701-B-100 funded by MCIN/AEI/10.13039/501100011033 and by “ERDF A way of making Europe" and by Instituto de Salud Carlos III, Subdirección General de Redes y Centros de Investigación Cooperativa, Spanish Network for Research in Infectious Diseases (REIPI RD16/0016) co-financed by the European Development Regional Fund ‘A way to achieve Europe’ and operative programme Intelligent Growth 2014-2020. Margalida Mateu and Alex González-Alsina are the recipient of FPI fellowships from Comunitat Autonoma de les Illes Balears (CAIB).

## Conflict of Interest

The authors declare that the research was conducted in the absence of any commercial or financial relationships that could be construed as a potential conflict of interest.

## Publisher’s Note

All claims expressed in this article are solely those of the authors and do not necessarily represent those of their affiliated organizations, or those of the publisher, the editors and the reviewers. Any product that may be evaluated in this article, or claim that may be made by its manufacturer, is not guaranteed or endorsed by the publisher.
