# Supplementary material for: Molecular Analysis of the Contribution of Alkaline Protease A and Elastase B to the Virulence of Pseudomonas aeruginosa Bloodstream Infections
Source: Front Cell Infect Microbiol. 2022 Jan 12;11:816356. doi: 10.3389/fcimb.2021.816356 (PMC8823171; doi:10.3389/fcimb.2021.816356)
Supplement: Supplementary file 1 [file DataSheet_1.docx]

**Supplementary Figure S1.** **Effect of the exoproteases AprA and LasB on the C3-cleaving activity of *P. aeruginosa***

Representative Western blot analysis of C3 cleavage by *P. aeruginosa*. (A) Purified human C3 (300 ng) or (B) human serum (10%) were incubated for 3 h at 37ºC with LB (control) or the cell-free supernatants obtained from cultures of the wild-type (WT) *P. aeruginosa* PA14 and the isogenic deficient mutants in AprA (Δ*aprA)*, LasB (Δ*lasB)* or both (Δ*aprA* Δ*lasB*). Proteins were separated by SDS-PAGE and subjected to a Western blot with a rabbit polyclonal antibody anti-C3. Specific cleavage of the C3α chain (black arrow) resulted in release of a ≈100 kDa product, C3α" (white arrow). Grey arrow indicates the β -chain of C3.

**Supplementary Figure S2. Impact of AprA and LasB on the virulence of *P. aeruginosa* in the *C. elegans* and *G. mellonella* models.**

A) Survival curves over 7 days of *C. elegans* (n = 5 per strain) infected with *P. aeruginosa* WT strain PA14 (orange) or its derived isogenic mutants PA14Δ*aprA* (green)*,* PA14Δ*lasB* (yellow) and PA14Δ*aprA*Δ*lasB* (blue). The data represents the mean of three independent experiments. Error bars represent SEMs.

B) LD_50_ of *P. aeruginosa* WT strain PA14 or its derived isogenic mutants in the *G. mellonella* model. Different doses (10, 5 and 2 CFU) were injected to groups of 10 larvaes per dose and strain. After 24 h, the mortality rate was determined. The LD50 was calculated using the Probit´log(dose) regression model. Data represents the mean of three experiments. Errors bars represent SEMs.

**Supplemetary Figure S3. Analysis of the expression of *aprA* and *lasB* .**

Relative expression of *aprA* and *lasB* in PA14 grown in heat inactivated serum (white columns) at different time points. Data represents three experiments done in duplicate. Errors bars represent SEMs.


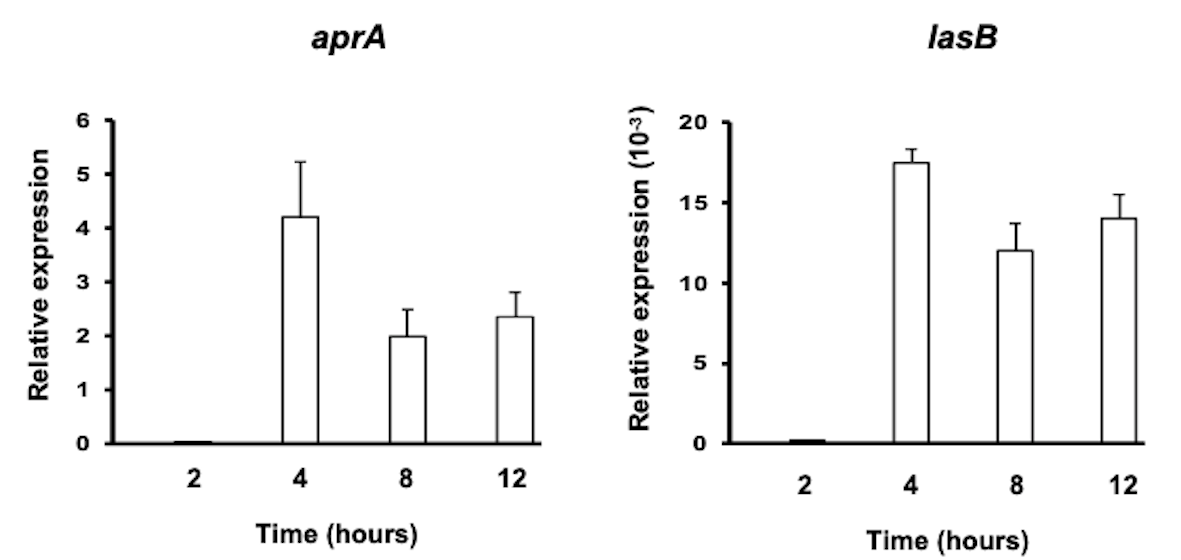


**Supplementary Figure S4. Impact of AprA and LasB on the survival of *P. aeruginosa* in mouse blood.**

Quantitative cultures of *P. aeruginosa* strains recovered from blood of mice 12 and 24 h after intraperitoneal challenge with 5×10^5^ CFU of *P. aeruginosa* WT strain PA14 (orange), or its derived isogenic mutants PA14Δ*aprA* (green)*,* PA14Δ*lasB* (yellow) and PA14Δ*aprA*Δ*lasB* (blue).

**Supplementary table S1.** Primers used in this study.

| Primer | Sequence (5’-3’) |
| --- | --- |
| AprA-Forward | TCGGTGATGAGCTACTGGGA |
| AprA-Reverse | AGAAGTCCAGGGTGTCGTTG |
| LasB-Forward | TGTCCAAACTCCCCAGCAAG |
| LasB- Reverse | GCGATGTTGGCGACGAAATG |
| Rpsl-Forward | GCTGCAAAACTGCCCGCAACG |
| Rpsl- Reverse | ACCCGAGGTGTCCAGCGAACC |
